# High-Grade Undifferentiated Pleomorphic Sarcoma of the Distal Tibia Presenting as a Radiographically Benign-Appearing Lesion: A Case Report and Review of the Literature

**DOI:** 10.3390/diagnostics16182971

**Published:** 2026-09-14

**Authors:** Carlo Biz, Elisa Pagliarini, Lorenzo Costa, Antonella Russo, Giuseppe Di Rubbo, Giulia Trovarelli, Pietro Ruggieri

**Affiliations:** 1 Department of Surgery, Oncology and Gastroenterology DiSCOG, University of Padova, Via Giustiniani 3, 35128 Padova, Italy; elisa.pagliarini@aopd.veneto.it (E.P.); lorenzo.costa@aopd.veneto.it (L.C.); antonella.russo.1@studenti.unipd.it (A.R.); giuseppe.dirubbo@studenti.unipd.it (G.D.R.); giulia.trovarelli@unipd.it (G.T.); pietro.ruggieri@unipd.it (P.R.); 2 Department of Orthopedics and Orthopedic Oncology, Padua University—Hospital, Via Giustiniani 3, 35128 Padova, Italy; 3 Centre for Mechanics of Biological Materials, University of Padova, 35131 Padova, Italy

**Keywords:** undifferentiated pleomorphic sarcoma, bone sarcoma, distal tibia, osteolytic lesion

## Abstract

**Background:** Undifferentiated pleomorphic sarcoma (UPS) of the bone is a rare, high-grade malignancy of uncertain origin, most commonly affecting the extremities of adults. Its diagnosis may be challenging, particularly when clinical and radiological findings are confounded by trauma-related conditions. **Case presentation:** In this paper, the case of a 51-year-old man who sustained a high-energy trauma resulting in a diaphyseal fracture of the left tibia and fibula, initially treated with intramedullary nailing. Preoperative radiographs raised suspicion of an osteolytic lesion in the distal tibia; however, second-level imaging was performed postoperatively, suggesting a benign process. At initial evaluation at a primary Musculoskeletal Oncology Centre, strict clinical and radiological follow-up was recommended, but the patient failed to adhere. Over the following years, persistent symptoms were attributed to post-traumatic and inflammatory conditions, including suspected complex regional pain syndrome. Progressive clinical deterioration led to repeat imaging, revealing an aggressive lesion. A biopsy (March 2025) ultimately confirmed a primary bone high-grade UPS with no metastasis. Following multidisciplinary discussion, below-knee amputation was performed, achieving clear surgical margins. At one-year CT imaging follow-up, the patient remained disease-free and was able to ambulate with a prosthesis. **Conclusions:** High-energy trauma and later attribution of symptoms to post-traumatic conditions may complicate the diagnostic process, contributing to diagnostic delay. Early referral to specialised Musculoskeletal Oncology Centres, strict adherence to follow-up, and timely histological confirmation are essential to optimise outcomes. Suspicious bone lesions should always be fully characterised prior to surgical intervention.

## 1. Introduction

Undifferentiated pleomorphic sarcoma (UPS) is one of the most common high-grade sarcomas in adults, although it remains a relatively rare malignancy of uncertain histogenesis [1]. It is important to distinguish soft tissue UPS from primary UPS of bone, an exceedingly uncommon and aggressive bone sarcoma without a specific differentiation line or pattern representing approximately 3% to 8% of all bone tumours [2]. Moreover, bone UPS predominantly affects older adults, occurring between the fourth and the seventh decade of life, with a peak incidence between the fifth and seventh [1,2,3,4,5,6]. Nevertheless, this condition can appear at any age, with a slight prevalence for males [1,2,4,6].

The clinical presentation of UPS is often insidious, usually presenting as a painless swollen limb and limitation of movement with variable growth rates [6]. It most commonly affects the lower extremities (particularly the thigh), as well as the upper extremity [3,6]. Rarely, systemic manifestations occur, including leukocytosis (with neutrophilia or eosinophilia) and hypoglycaemia. Unintentional weight loss and fatigue are also uncommon [7,8].

Plain radiography is the first-line imaging modality and, with the Modified Lodwick–Madewell classification, provides valuable information on lytic bone patterns and lesion margins, thereby helping to estimate tumour aggressiveness [9]. Lesions are classified as Grade I, characterised by low-risk features and well-defined geographic margins; Grade II, displaying intermediate-risk features with ill-defined, non-sclerotic margins. Lastly, Grade III is characterised by high-risk features with poorly defined margins and permeative or moth-eaten patterns of bone destruction [10,11].

When a primary malignant bone tumour is suspected, MRI represents the preferred modality for local staging, allowing assessment of intramedullary tumour extent, cortical involvement, extraosseous extension, and relationships with the surrounding neurovascular structures [7,12]. Systemic staging should include chest CT as the lungs represent the most frequent metastatic site in primary bone sarcomas. Definitive diagnosis requires histological confirmation, preferably by image-guided core needle biopsy, which should be carefully planned within a multidisciplinary sarcoma setting to minimise the risk of contaminating disease-free anatomical compartments and to ensure that the biopsy tract can be encompassed within the subsequent definitive surgical resection.

Moreover, it is important to have an experienced anatomopathologist in the multidisciplinary team, as it can be often misled as chondrosarcoma [5]. Treatment for primary UPS of bone necessitates a multidisciplinary team, including surgery and radiation, achieving partial tumour response in approximately 50% of cases and is frequently employed preoperatively to reduce cancer volume and facilitate radical surgical resection. Adjuvant chemotherapy is often used as part of the osteosarcoma protocol, and it has been associated with improved survival outcomes [1,3,4,6,13,14].

Primary bone UPS is an aggressive malignancy, with prognosis mainly influenced by tumour size, surgical margins, and the presence of metastatic disease at diagnosis [15]. In contrast, the frequently reported local recurrence rates of approximately 20–30% mainly derive from soft-tissue UPS series and should not be directly extrapolated to primary bone UPS [1,6,7,15]. The five-year survival rate for UPS is 65–70%, with approximately 35% of cases experiencing metastasis [2,15]. Nevertheless, probably due to its rarity, tumour located in distal regions such as the ankle appears to have a worse prognosis compared to proximal lesions [7,15,16,17]. As a matter of fact, the available literature remains scarce, with only a limited number of cases reported to date, and the optimal management of this rare presentation remains controversial. Therefore, despite its rarity, raising awareness of primary bone UPS and providing a clearer synthesis of the available evidence are important to support timely diagnosis and appropriate clinical management.

Hence, the present study aims to report the case of an adult male with primary UPS of bone of the distal tibia, initially presenting as an incidental, benign-appearing osteolytic lesion in the setting of high-energy trauma. Beyond describing this rare clinical presentation, this report highlights the potential diagnostic and therapeutic consequences of undertaking definitive orthopaedic treatment, particularly intramedullary fixation, before an unexpected bone lesion has been adequately characterised. Through this case and an updated review of the literature, we further emphasise the importance of appropriate oncological imaging, carefully planned biopsy when indicated, and early referral to a specialised sarcoma or orthopaedic oncology centre whenever the nature of a bone lesion remains uncertain.

## 2. Case Presentation

**Figure 1:**
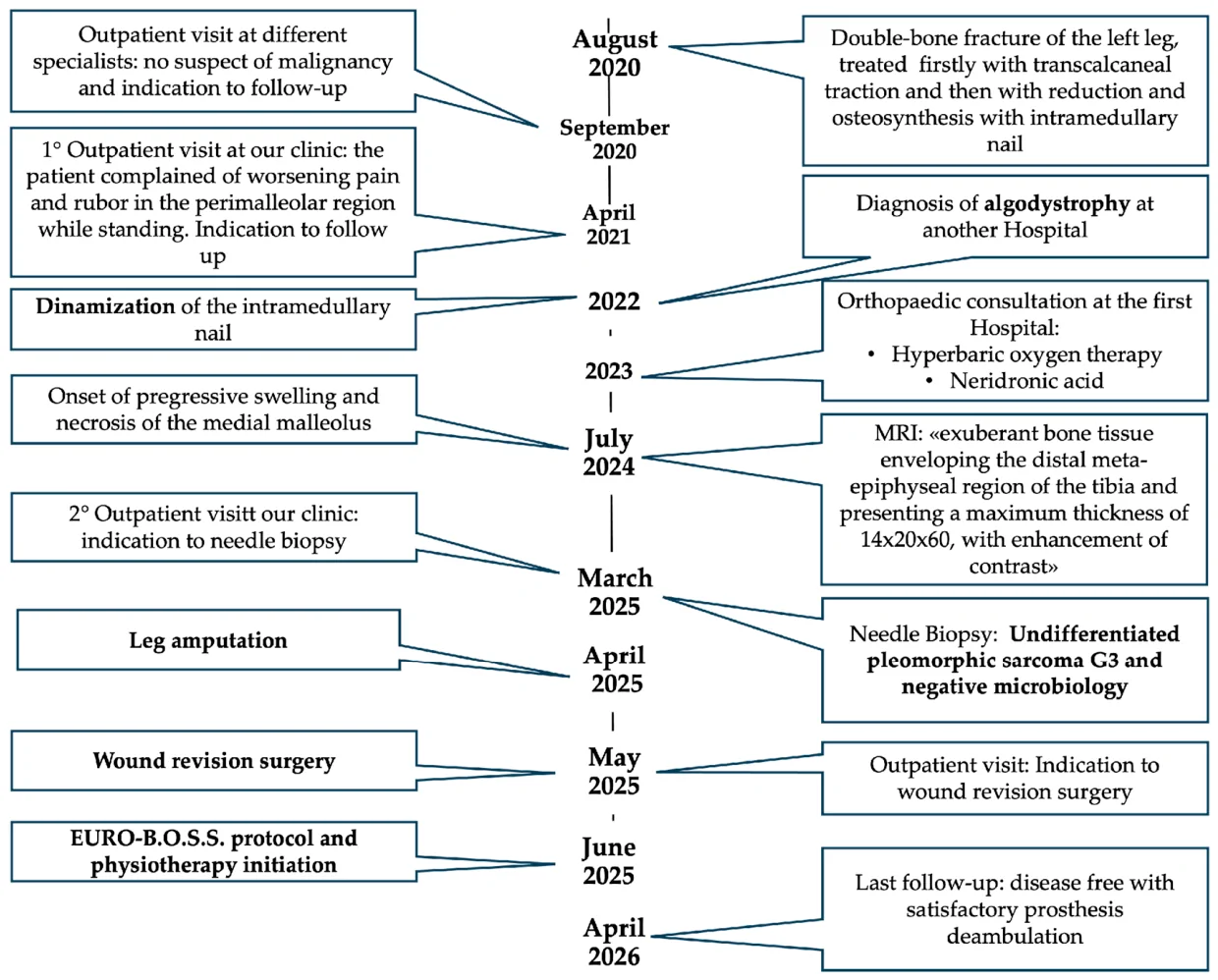
Flow chart of the case report: timeline of patient management from the initial traumatic event to lower-limb prosthesis fitting.

This case report was described in accordance with the Consensus-based Clinical Case Reporting Guidelines as published in 2014, and following the Flow Diagram for Case Reports as updated in 2017 (Figure 1) [18,19].

A 51-year-old man, previously healthy and with no prior history of malignancy, was involved in a high-energy road traffic accident in August 2020 after being struck by a car. He reported a displaced diaphyseal fracture of both tibia and fibula of the left leg with a small tibial bone exposition classified as Gustilo-Anderson type I. Pre-operative plain radiographs also revealed an osteolytic lesion, prompting the radiologist to recommend further evaluation with advanced imaging. The lesion exhibited a geographic pattern of bone destruction with well-defined margins and no radiographic features typically associated with aggressive malignancy, such as cortical permeation, moth-eaten pattern of bone destruction, or aggressive periosteal reaction [10]. At this stage of the diagnostic work-up, the distal tibial lesion therefore appeared radiologically non-aggressive and was classified as Grade IA according to the Modified Lodwick–Madewell classification [11].

**Figure 2:**
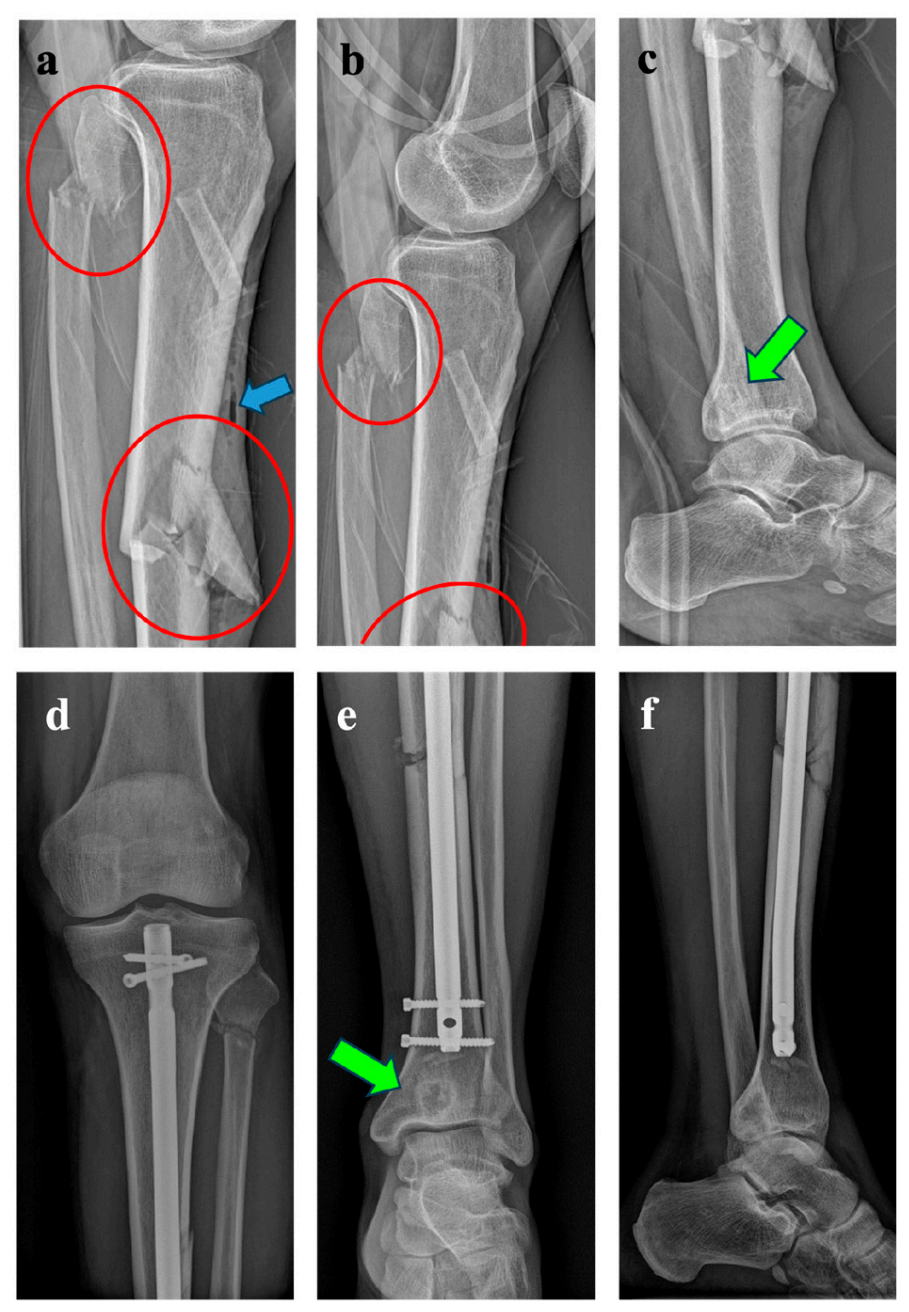
Preoperative radiographic images (**a**–**c**) demonstrating a proximal fibular diaphyseal fracture and a mid-diaphyseal fracture of the tibia, highlighted by red circles. Postoperative radiographs (**d**–**f**) following external reduction and intramedullary nail fixation. The blue arrow indicates the site of exposure in (**a**) (Gustilo type I). The green arrow highlights an osteolytic lesion (Grade IA Lodwick and Madewell), visible on both preoperative (**c**) and postoperative (**e**) radiographic images.

Despite the radiologist’s recommendation for further imaging evaluation, initial management in the emergency department consisted of trans-calcaneal skeletal traction, followed the next day by definitive fracture reduction and intramedullary nailing at the orthopaedic department of the same institution in the patient’s city of residence. (Figure 2).

**Figure 3:**
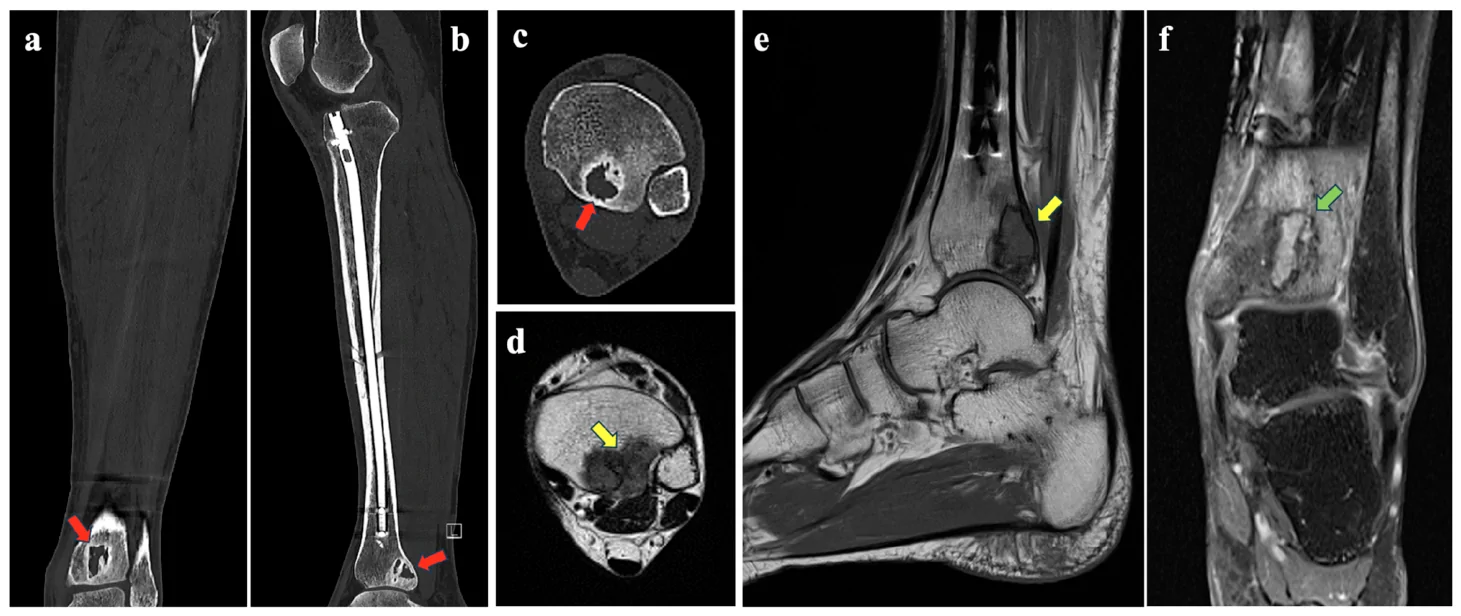
Postoperative CT and MRI performed in September 2020. Post-operative CT (**a**–**c**), showing a well-circumscribed lytic lesion in the distal tibia with a non-aggressive appearance, classified as Grade IA according to the Modified Lodwick–Madewell classification (red arrows). Post-operative MRI images performed without contrast enhancement showing the same lesion in the distal tibia with a low signal in T1 sequences (yellow arrows) and not well-defined borders (**d**,**f**); and high signal in T2 sequences with an intense surrounding peri-lesional bone marrow oedema (green arrow) (**e**).

Non-contrast-enhanced CT and MRI were performed only during the immediate postoperative period in September 2020 at the same Orthopaedic Department and confirmed the presence of an osteolytic lesion in the distal tibia with benign-appearing features. (Figure 3).

As no specific diagnostic work-up, management, or treatment had been recommended for the lesion, the patient independently sought a second opinion at a specialised musculoskeletal oncology centre.

**Figure 4:**
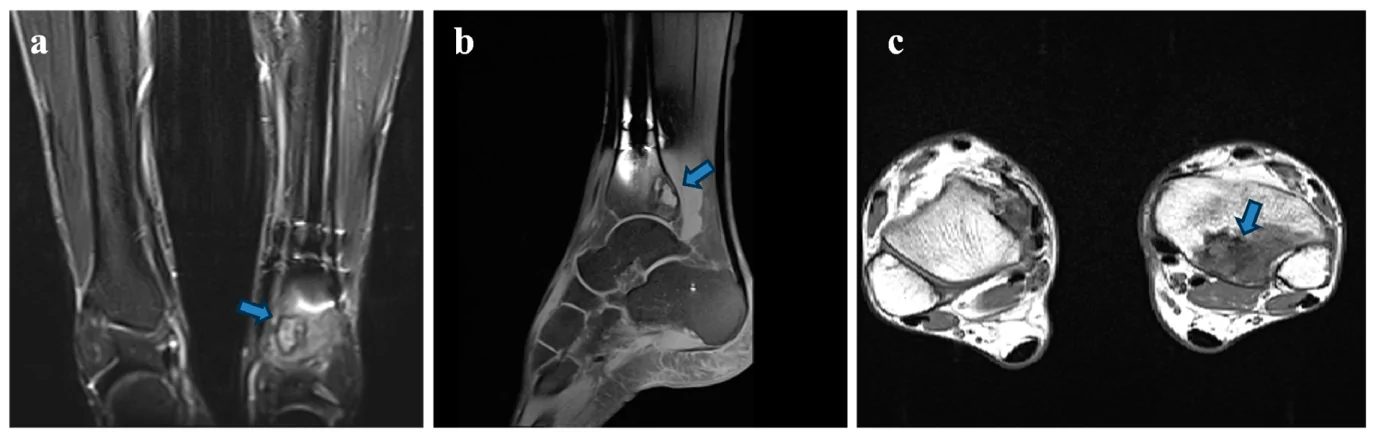
Contrast-enhanced MRI performed in April 2021 demonstrating the lesion (blue arrows): post-contrast enhancement on the coronal plane (**a**), features suggestive of high cellularity on Dixon sequences in the sagittal plane (**b**), and a poorly defined lesion with low signal intensity on T1-weighted axial images compared with the contralateral distal leg (**c**).

The patient first presented to the outpatient clinic of our Orthopaedic Department in April 2021, when a new radiological assessment was performed, including an updated contrast-enhanced MRI (Figure 4). However, the lesion remained insufficiently characterised. Although the initial imaging findings in September 2020 had suggested a benign-appearing lesion (less aggressive subgroup IA), the 2021 MRI revealed more concerning features, including marked intraosseous and perilesional bone marrow oedema. These findings warranted close imaging surveillance and further diagnostic investigation [11].

Therefore, an updated contrast-enhanced CT scan was recommended to reassess the lesion and determine whether additional diagnostic investigations, including biopsy, were required. However, the patient did not complete the recommended investigations and was subsequently lost to follow-up. He returned to our centre only three years and eleven months later.

In January 2022, while under the care of the primary treating centre, the patient developed progressive peri-malleolar pain and erythema, which exacerbated during weight-bearing; dynamisation of the intramedullary nail was therefore performed. Further, in 2023, the patient was treated for suspected complex regional pain syndrome (CRPS) with hyperbaric oxygen therapy and neridronate, resulting in partial clinical improvement.

**Figure 5:**
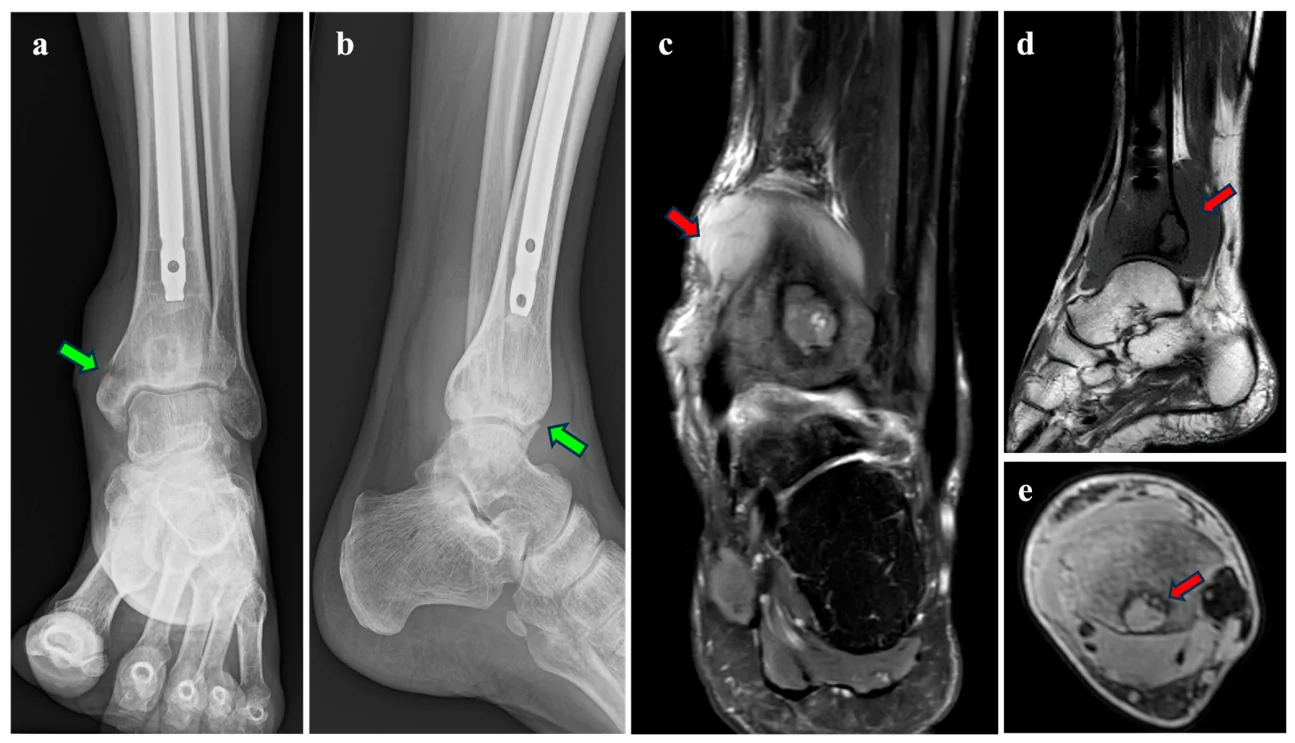
Anteroposterior and lateral radiographs obtained in July 2024 demonstrating a well-circumscribed lytic lesion associated with a pathological fracture of the medial malleolus (green arrow), as well as the intramedullary nail following screw removal for dynamisation (**a**,**b**). Contrast-enhanced MRI revealed enlargement of the distal tibial lesion compared with previous imaging, with a prominent soft-tissue component visible on all sequences (**c**–**e**) showing post-contrast enhancement (**c**), low signal intensity on T1-weighted images (**d**), and marked perilesional bone marrow oedema on T2-weighted sequences (**e**) (red arrows).

In July 2024, because of progressive painful swelling, plain radiographs of the left ankle were obtained and raised suspicion of medial malleolar bone necrosis associated with a pathological fracture (Figure 5a,b). Consequently, a new contrast-enhanced MRI of the left ankle was performed (Figure 5c,d), demonstrating an extensive abnormal tissue component surrounding the distal tibial meta-epiphyseal region, measuring up to 14 × 20 × 60 mm and showing marked contrast enhancement.

Following further outpatient evaluation at the original treating hospital, the patient was referred to our centre for a second time in March 2025. Given the differential diagnosis of a cartilaginous neoplasm versus chronic infection, fluoroscopy-guided needle biopsies were performed shortly thereafter, targeting both the osseous component of the distal tibial lesion and the peri-malleolar soft-tissue component.

Histological examination of the peri-malleolar soft-tissue and distal tibial bone biopsies revealed a malignant mesenchymal neoplasm composed of spindle and pleomorphic cells, infiltrating both the fibro-ligamentous tissue and bone trabeculae. Immunohistochemistry showed focal positivity for smooth muscle actin and negativity for desmin, caldesmon, S100, MDM2, TCG, and cytokeratin MNF116, supporting the diagnosis of grade 3 UPS of the bone according to the FNCLCC grading system, whereas microbiological cultures were negative.

Baseline staging (total body CT scan) showed no evidence of distant metastatic disease. However, given the extensive local tumour involvement demonstrated on imaging (14 × 20 × 60 mm), with involvement of the distal tibia and ankle region, substantial extraosseous soft-tissue extension, close involvement of the neurovascular structures, and potential contamination of the tibial medullary canal following previous intramedullary nail insertion through the lesion, limb-sparing surgery was not considered oncologically feasible.

**Figure 6:**
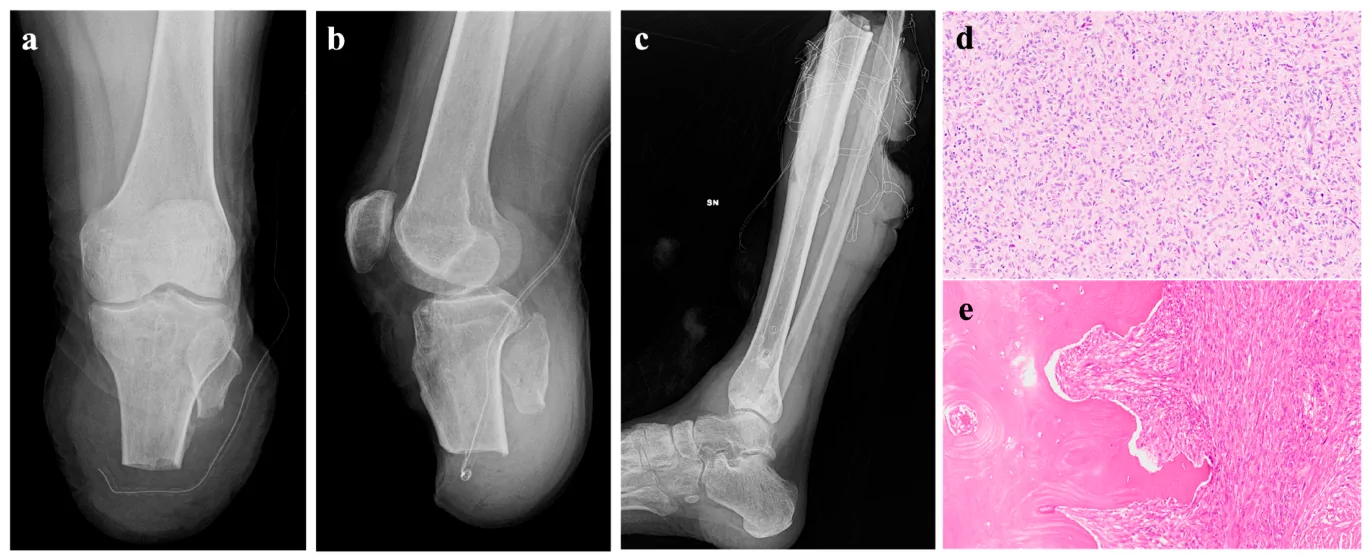
Radiographic images obtained on 22 April 2025 showing the left lower limb following below-knee amputation. (**a**,**b**) and cross-section of the amputated limb (**c**). Histological sections demonstrating tumour-free resection margins and a negative regional lymph node (**d**,**e**).

Due to extensive local disease, a multidisciplinary team recommended prompt definitive surgery without preoperative chemotherapy. Therefore, a below-knee amputation was performed on 22 April 2025. Final histological examination confirmed the diagnosis of high-grade UPS, with clear surgical margins and a regional lymph node incidentally included in the surgical specimen and examined histologically, which was negative for metastatic involvement. (Figure 6a,b).

The amputation specimen revealed a FNCLCC Grade 3 UPS, infiltrating the skeletal muscle and both the cortical and trabecular bone of the tibia (a left lower-limb specimen comprising a 37-cm segment of the lower leg and a 25.5-cm foot. At the level of the medial malleolus, an area of swelling and increased consistency, measuring 6.5 × 8 cm, was present, located 25 cm from the proximal resection margin. On sectioning, the peri-tibial soft tissues corresponding to the area of swelling were involved by a discoloured neoplastic lesion measuring 8 × 6.5 × 2 cm, which appeared to macroscopically infiltrate the tibia. The fibula showed no macroscopically evident neoplastic infiltration.

Moreover, the immunohistochemical work-up included MDM2, SATB2, smooth muscle actin (SMA), desmin, caldesmon, Myf4, S100, and cytokeratin MNF116. MDM2, SATB2, desmin, caldesmon, Myf4, S100, and cytokeratin MNF116 were negative, while SMA showed multifocal positivity (*Immunohistochemical profile: MDM2: negative; Smooth muscle actin (SMA): multifocal positivity; Desmin: negative; Caldesmon: negative; Myf4: negative; SATB2: negative; S100: negative; Cytokeratin MNF116: negative*). Overall, these findings confirmed the previous biopsy diagnosis of a high-grade UPS of bone, with no evidence of a specific line of differentiation. (Figure 6c–e).

**Figure 7:**
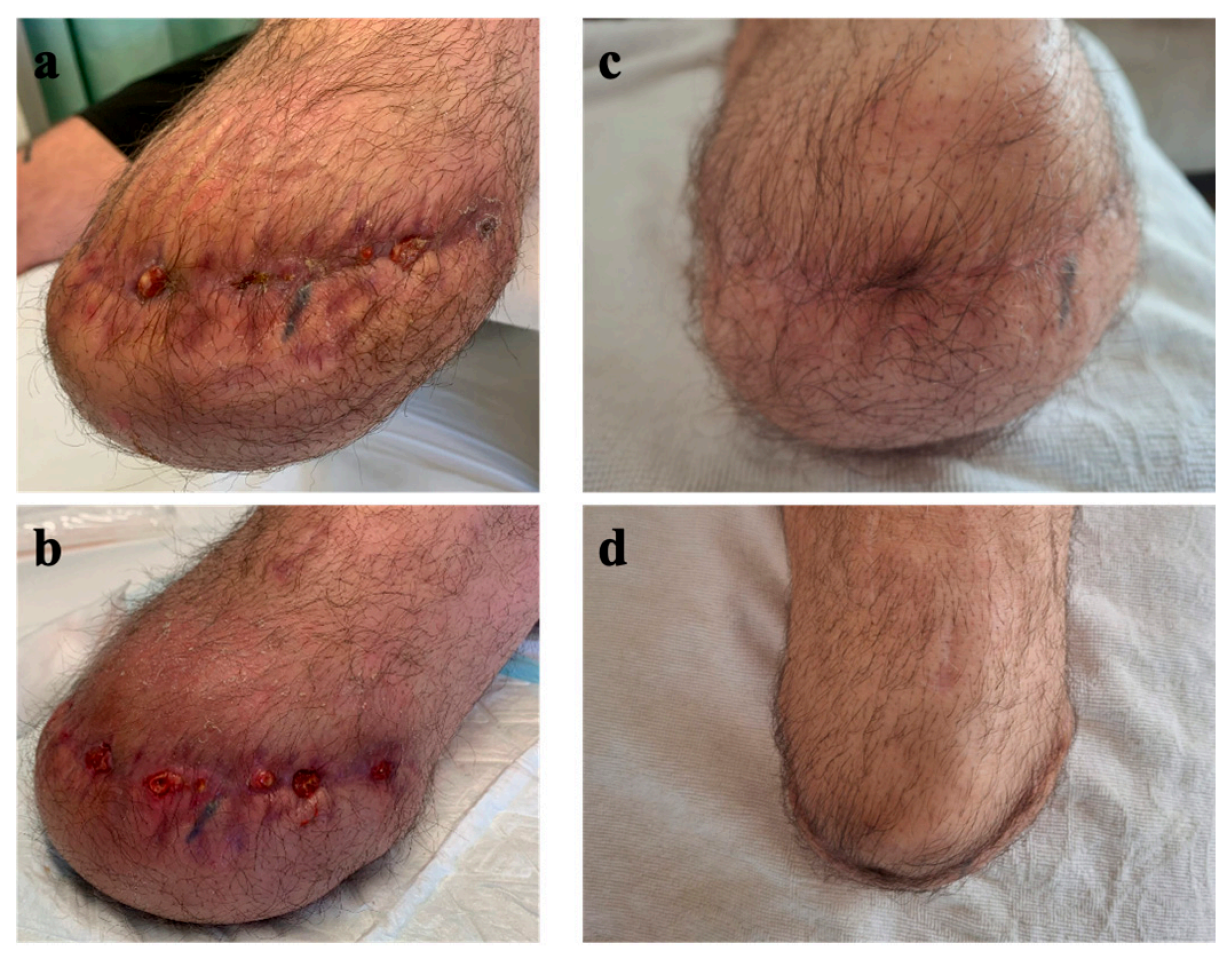
Clinical images demonstrating wound complications (**a**,**b**) and the healing after surgical revision and prior to prosthetic fitting (**c**,**d**).

At follow-up in May 2025, wound complications were identified, and surgical revision was performed the following days (Figure 7a,b).

Considering the patient’s clinical condition and performance status (weight: 90 kg; height: 186 cm; BMI: 26 kg/m^2^; Karnofsky Performance Status: 80%; ECOG Performance Status: 1), the EURO-B.O.S.S. regimen was selected. This protocol was specifically developed for patients aged >40 years with high-grade primary bone sarcomas, including UPS, and incorporates cisplatin (CDDP, 100 mg/m^2^), doxorubicin (ADM, 60 mg/m^2^), and ifosfamide (IFO, 6 g/m^2^) [9]. Nine cycles of adjuvant chemotherapy were initially planned. However, the postoperative wound dehiscence resulted in a substantial delay in the initiation of systemic treatment. Given the prolonged interval from surgery, the multidisciplinary team considered that the expected benefit of initiating adjuvant chemotherapy at such a late stage would be limited, and the planned treatment was therefore not commenced. The patient was instead managed with close surveillance, including staging investigations every two months.

**Figure 8:**
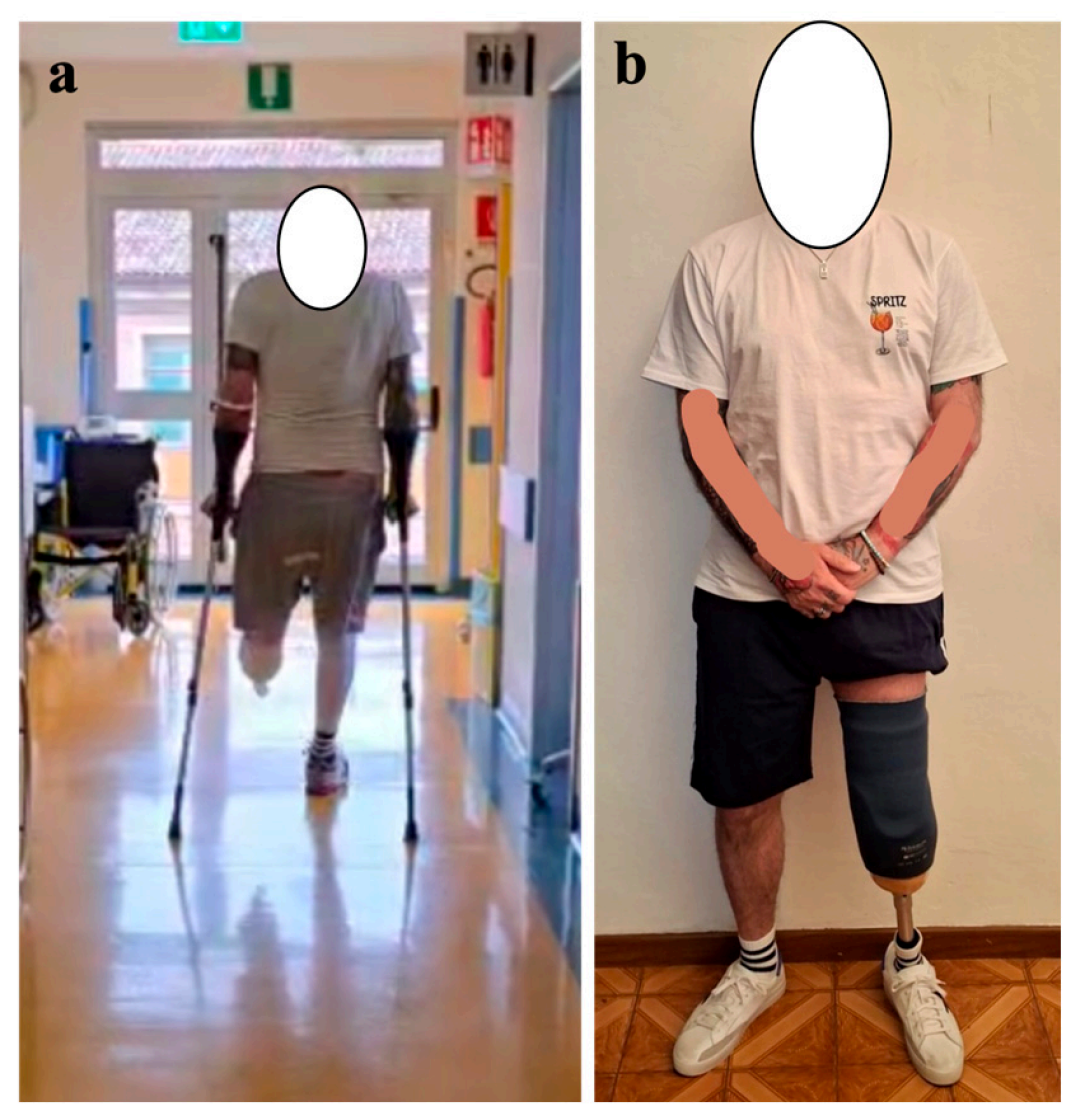
Clinical images demonstrating initiation of the rehabilitation program prior to prosthetic fitting (**a**); and patient at final follow-up with the prosthesis in situ, well tolerated (**b**).

In June 2025, once satisfactory wound healing had been achieved (Figure 7c,d), the patient started physiotherapy for prosthetic fitting (Figure 8a). At the one-year follow-up after surgery, in April 2026, the patient remained disease-free and was able to ambulate independently with the prosthesis (Figure 8b). He did not require walking aids and reported no significant pain or prosthesis-related discomfort, with no falls documented during the follow-up period, which remains ongoing at the time of writing.

## 3. Literature Review

A narrative literature search was performed to identify studies reporting UPS involving the lower extremities. A comprehensive literature search of the PubMed database was conducted up to April 2026 using search terms for primary bone UPS of the extremities, including case reports, case series, retrospective studies, and larger clinical cohorts focused on clinical presentation, diagnostic work-up, surgical strategies and margins, metastatic disease, recurrence, and clinical outcomes.

Eaton et al. described a case report with high-grade UPS of the tibia that initially mimicked a benign lesion on ECO and CT scan, emphasizing the diagnostic challenges. Definitive diagnosis required an MRI scan with contrast and needle core biopsy. CT scan of the body was subsequently done to study possible metastasis sites, which luckily resulted in negative. The patient was successfully treated with a multidisciplinary approach, including embolisation of three branches of the anterior tibial artery which supplied the mass, wide surgical resection with R0 margins, and complex soft tissue reconstruction of the right calf. The patient was discharged 14 days after surgery and no subsequent follow-up was performed. Although prolonged follow-up should be pivotal for the understanding of the success of the treatment, the current study highlights the importance of accurate diagnosis and the necessity of a multidisciplinary approach involving a prepared radiologist, anatomopathologist, oncologist and orthopaedic surgeon [20].

Bielack et al. analyzed 132 patients with undifferentiated pleomorphic sarcoma of bone (UPSB), 86% (n = 114) of which affected the extremities and 24 of them the tibia (proximal 20, diaphysis 3, distal 1). All patients received multi-agent chemotherapy, and 96% of cases underwent primary surgery (81% via limb-salvage for extremity lesions). A good histological response to preoperative chemotherapy was observed in 38% of evaluable cases. The 1-, 2-, 5-, and 10-year overall survival and event-free survival probabilities were 95% (standard error: 2%), 86% (3%), 70% (4%), 64% (5%) and 88% (3%), 78% (4%), 63% (5%), 55% (5%), respectively. In multivariable analysis, older age, primary non-extremity tumor site, and the presence of primary distant metastases were confirmed as independent predictors of worse survival. Conversely, histological response to preoperative chemotherapy did not demonstrate an independent prognostic impact on survival [21].

Gonzalez et al. in their large retrospective study confirm that UPS of the extremities is frequently diagnosed as large tumours, with size ≥ 7 cm significantly worsening survival. Metastasis, particularly to the lungs, and lymph node involvement are major predictors of poor outcome, while local recurrence remains a significant risk. Nevertheless, distal tibia was not analysed separately, and differences between the sites were not available [15].

Zhang et al. 2022 performed a retrospective study with a mean follow-up of 55 months, revealing that the UPS in the trunk, tumour size ≥ 5 cm and R1/R2 resection margin are prognostic markers of poor survival rate [22].

An interesting report written by Huff et al. showed the complex case of an 81 y.o. woman with progressive hip pain. Physical examination and subsequent sacro-lumbar x-ray and MRI revealed disc degeneration at L2–L3 and L3–L4, causing moderate foraminal stenosis at L2–L3. Her pain was initially attributed to these disc herniations. After 2 months the patient began having gait difficulties and noted an enlarging mass at her right thigh. MRI of the hip revealed a multicystic mass measuring up to 13.7 × 12.3 × 38.6 cm, which encircled her sciatic nerve. Subsequent needle core biopsy of the mass confirmed the diagnosis of UPS. A F18 PET-CT showed hypermetabolic lung lymph nodes, and successive bronchoscopy with wedge resection confirmed tumour infiltration. Palliative radiotherapy was thus performed, and systemic chemotherapy was attempted, resulting in the exitus of the patient 4 months after diagnosis and 6 months after symptoms began [23].

Alpert et al. described a case of a 48 y.o. man presenting with a rapidly growing anterior calf mass. Initial aspiration by a dermatologist was attempted, with no symptom regression. Subsequent MRI and biopsy revealed UPS infiltrating the subcutaneous tissue and fascial planes. An initial marginal excision with positive margins was performed by a general surgeon. Re-resection followed by radiotherapy (total amount of 63 Gy) was thus performed. Despite this, a subtle enhancing tissue along the fascia was found but it was initially interpreted as postoperative change. Over more than 7 years, this lesion demonstrated slow, but consistent growth. Biopsy was thus performed, and it confirmed local recurrence of UPS. The patient underwent a complex reconstructive surgery using an anterolateral thigh free flap and split-thickness skin graft were successfully performed. At the last available follow-up at 15th month after surgery was still disease-free [24]. Table 1 shows the papers that have been included in the review.

## 4. Discussion

This case illustrates a prolonged diagnostic pathway, from the initial trauma and fracture fixation in August 2020 to the diagnosis of high-grade primary bone UPS in March 2025, despite progressive pain, swelling, and radiological changes that ultimately prompted biopsy and definitive treatment.

Undifferentiated pleomorphic sarcoma (UPS) of the bone represents one of the most aggressive and poorly differentiated malignant mesenchymal neoplasms, characterised by complex genomic alterations and a highly variable clinical course that demands sophisticated diagnostic and therapeutic strategies [25,26]. This rare malignancy, historically named malignant fibrous histiocytoma, typically arises in the extremities and trunk of older adults and carries a significant risk of local recurrence and distant metastasis despite multimodal interventions [26]. Primary bone involvement is uncommon, which inherently complicates the initial workup. Furthermore, presentation in the distal tibia is exceptionally rare, often positioning this entity as a diagnosis of exclusion [1,2,3,15,17,20,27,28].

This case report and its associated review highlight the risk of diagnostic pitfalls and potential consequences of delayed recognition of malignant pathology in the context of trauma and routine orthopaedic work-up. The incidental identification of an osteolytic lesion in the distal tibia at the time of the fracture should have raised immediate concern. A different treatment workflow, consisting of external fixation with MRI-compatible materials, would have given more time for accurate investigation. Therefore, further diagnostic evaluation with CT and MRI with and without contrast could have been performed prior to definitive surgical treatment to permit adequate identification of the lesion. While the initial radiographic features suggested a benign lesion (Grade IA/IB seemingly), the histological assessment and MRI performed at our centre highlighted malignant and high-grade bone sarcoma [11]. Although extremely rare for classic bone sarcomas, such radiographic underestimation of malignancy is a well-documented pitfall in other low-incidence bone tumours [10,29,30,31].

In retrospect, the incidental detection of a lytic bone lesion following a traumatic fracture in August 2020, despite its apparently benign Grade IA radiographic features, should have prompted further advanced imaging before definitive fracture fixation. Contrast-enhanced CT would have been mandatory in the trauma setting to better assess cortical involvement, matrix characteristics, and the relationship of the lesion to the fracture site, while MRI with contrast would have provided pivotal information on bone marrow extension and soft-tissue involvement. Based on these findings, referral to a musculoskeletal oncology centre and a preoperative biopsy should have been strongly suggested [10,30,31].

Furthermore, in the absence of a definitive pathological diagnosis, proceeding directly to intramedullary nailing carried the risk of contaminating the medullary canal and surrounding tissues, potentially compromising subsequent oncological treatment options [32]. This case therefore emphasises the importance of maintaining high suspicion whenever a fracture occurs through a lytic lesion, highlighting the need for a multidisciplinary diagnostic workup before definitive surgical intervention.

These investigations, although performed only in the postoperative period, were suggestive of a benign lesion, a diagnostic complexity also described by Eaton et al. and Huff et al., where UPS mimicked benign or degenerative conditions [20,23,24]. Accordingly, it was recommended that strict clinical and radiographic follow-up be performed at three-month intervals during the patient’s initial evaluation at our centre. However, follow-up was delayed due to communication barriers, and diagnostic anchoring on post-traumatic or inflammatory conditions may have delayed the UPS diagnosis [33,34]. While the tumour extended irreversibly into the distal leg and ankle, there was fortunately no metastasis. Furthermore, postoperative pain and erythema, as well as the suspicion of regional pain syndrome, due to the high-energy fracture likely led to bias by masking the malignancy as a post-traumatic or inflammatory process [35,36].

Nevertheless, generic MRI features marked heterogeneity on T1- and T2-weighted sequences reflecting the pleomorphic nature of the tumour, with signal intensity variations corresponding to areas of necrosis, haemorrhage, cystic degeneration, and viable tumour tissue, making UPS most likely an exclusion diagnosis [37,38]. Finally, definitive diagnosis was only possible after guided biopsy, as in this case, emphasizing that histopathological confirmation remains pivotal [3,8,12,20,21]. Image-guided biopsy is essential to ensure adequate material for comprehensive histological and molecular analysis while minimizing the risk of seeding along the needle track [39]. Indeed, to mitigate the risk of tumour spread, the biopsy must be carefully planned and marked, allowing the entire track to be excised en bloc with the tumour during definitive surgical resection [39,40].

The delay in diagnosis inherent to the indolent early clinical course of UPS has significant implications for prognosis, as many patients present with advanced locoregional disease or evidence of distant metastasis at the time of initial diagnosis [20]. Potential metastatic disease, which leads to poor outcomes, as described by Huff et al., where early metastatic spread led to rapid clinical deterioration [23]. According to Bielack et al., metastatic disease at presentation represents one of the strongest negative prognostic factors, suggesting that our patient may still have a relatively favourable prognosis despite the need for amputation due to extensive local infiltration [41]. Therefore, it is pivotal to perform CT scans of the body, mainly of the chest, as the lungs are the organs most easily affected [3,12,15,16,17,21]. This allows us to find possible distant locations of the disease, precisely define prognosis, and determine the most appropriate treatment strategy [21,22,23,24].

High-grade UPS carries a poor prognosis, with high rates of local recurrence and distant metastasis [1,3,7,15,17]. Radical surgical resection with clear margins remains the cornerstone of treatment [3,17,21,22]. The goal of surgical therapy is to completely excise the tumour with surrounding normal tissue while preserving maximal function, a principle encompassed in the concept of limb salvage surgery, which represents the standard of care for most patients with extremity sarcomas [3,22,42]. Wide resection requires meticulous surgical planning to account for compartmental anatomy, with determination of whether the tumour is confined within a single muscle compartment or has invaded multiple compartments, a distinction that fundamentally alters the extent of tissue requiring resection [3].

Compared to the large cohort reported by Bielack et al., distal tibial involvement remains exceedingly rare [21]. Their findings emphasise that extremity UPS generally carries a better prognosis due to the feasibility of achieving R0 resection (microscopically margin-negative resection) [21]. However, our case demonstrates that this advantage may be offset by delayed diagnosis and advanced local disease at presentation. In line with Gonzalez et al. and Zhang et al., tumour size and surgical margins are critical prognostic factors [15,22]. Moreover, unlike Eaton et al. and Alpert et al., our patient, following multidisciplinary discussion, necessitated limb amputation, given the involvement of surrounding tissues and the need to achieve adequate disease-free margins [20,24]. It is therefore important to notice that, although limb-sparing approaches are preferred, extensive resection and consequent reconstruction or amputation remain necessary in cases of extensive local involvement or when clear margins cannot otherwise be achieved [1,14,17,27,28,42].

Finally, definitive diagnosis was only possible after guided biopsy, as in the present case, emphasising that histopathological confirmation remains pivotal [3,8,12,20,21]. Image-guided biopsy is essential to obtain adequate material for comprehensive histological and molecular analysis while minimising the risk of seeding along the needle track [39]. Indeed, to mitigate the risk of tumour spread, the biopsy must be carefully planned and marked, allowing the entire biopsy tract to potentially be excised en bloc with the tumour during definitive surgical resection as happened in our case [39,40].

The delay in diagnosis may have negative prognostic implications, as some patients present with advanced locoregional disease or metastases at diagnosis as illustrated by Huff et al. [20,23]. Moreover, according to Bielack et al., metastasis at presentation represents one of the strongest negative prognostic factors [41]. In the present case, baseline total-body CT staging, with particular focus on the lungs as the most frequent site of metastasis, showed no evidence of metastatic disease [3,12,15,16,17,21]. Appropriate systemic staging allows the detection of distant disease and contributes to prognostic assessment and treatment planning [21,22,23,24].

High-grade UPS of the bone carries a poor prognosis, with high rates of local recurrence and distant metastasis [1,3,7,15,17]. Radical surgical resection with clear margins remains the cornerstone of treatment [3,17,21,22]. The goal of surgical therapy is to achieve complete tumour excision with adequate margins while preserving maximal function whenever oncologically feasible, according to the principles of limb-salvage surgery [3,22,42]. Wide resection requires meticulous surgical planning based on tumour extent and compartmental anatomy [3]. In the present case, however, the distal tibial meta-epiphyseal lesion measured 14 × 20 × 60 mm and involved the ankle region, with substantial extraosseous soft-tissue extension and close involvement of the adjacent neurovascular structures. Moreover, the previous passage of the intramedullary nail through the lesion raised concern for contamination of the tibial medullary canal. When UPS arises in critical anatomical locations with extensive involvement of neurovascular structures, or when adequate surgical margins cannot be achieved while preserving vital function, amputation or radical disarticulation may be required [1,14,17,27,28].

Compared with the large cohort reported by Bielack et al., distal tibial involvement remains exceedingly rare [21]. Their findings emphasise that extremity UPS generally carries a better prognosis due to the greater feasibility of achieving an R0 resection (microscopically margin-negative resection) [21]. However, in the present case, delayed diagnosis was associated with advanced local disease at presentation. In line with Gonzalez et al. and Zhang et al., tumour size and surgical margins are critical prognostic factors [15,22]. In contrast to what was reported by Eaton et al. and Alpert et al., following multidisciplinary evaluation, limb-sparing surgery was not considered oncologically feasible in our patient due to the extensive involvement of the distal tibia and ankle region, extraosseous soft-tissue extension to the neurovascular structures, and potential contamination of the tibial medullary canal by the previous intramedullary nail [20,24]. Although limb-sparing approaches are preferred whenever feasible, extensive resection with reconstruction or amputation may remain necessary when adequate tumour-free margins cannot otherwise be achieved [42].

This case report has some limitations. First, by its very nature, a single case provides only a limited perspective on the disease, as each patient may present with unique clinical and biological characteristics. Second, the absence of long-term follow-up restricts a comprehensive assessment of the patient’s sustained outcomes and long-term disease-free status. Therefore, the current case report gives only limited information about possible UPS re-presentation and long-term outcome. It is therefore important to state that the patient needs further follow-up over the next years for proper management. Third, the reconstruction of the case might have been constrained by inter-hospital data discrepancies and inconsistent imaging timelines. Finally, the literature review was heterogeneous and non-systematic, and no validated functional outcome measures were used to assess the patient’s clinical outcomes.

Nevertheless, the primary aim of this manuscript was to emphasise that accurate characterisation and diagnosis of any bone lesion or soft-tissue mass should precede definitive fracture management, thereby minimising the risk of inappropriate treatment and potentially adverse clinical outcomes, as illustrated by the present case. Conversely, the present study highlights several important clinical lessons. First, incidental identification of osteolytic or atypical bone lesions even during trauma workup requires close attention, and an advanced imaging diagnostic workflow with early CT and/or MRI should be performed. This would eventually be followed by a biopsy when uncertainty persists. Second, persistent or progressive pain, swelling or radiological changes in the presence of orthopaedic implants should not be attributed solely to post-traumatic sequelae or inflammatory syndromes and thoughtful investigation should be performed. Third, when there is suspicion of malignancy, early referral to specialised Musculoskeletal Oncology Centres endowed with a multidisciplinary team is pivotal to optimise diagnostic accuracy and treatment outcomes, as shown also in the literature [3,7,32].

## 5. Conclusions

In conclusion, the present case illustrates the diagnostic challenges associated with a rare distal tibial presentation of primary bone UPS, particularly when an unexpected bone lesion is identified in the setting of trauma. This case highlights the pivotal role of specialised musculoskeletal oncology centres and the importance of appropriately planned biopsy when imaging findings raise suspicion of a neoplastic process.

When a suspicious or indeterminate bone lesion is incidentally identified during trauma assessment, appropriate imaging should be undertaken to characterise the lesion before definitive surgical fixation, whenever clinically feasible. This approach should not delay emergency trauma stabilisation when clinically required; however, definitive instrumentation should preferably be deferred until malignancy has been reasonably excluded.

Finally, a multidisciplinary diagnostic work-up, effective communication among treating teams and with the patient, continuity of care, structured clinical and radiological follow-up, and timely histological assessment, when indicated, are essential to minimise the risk of diagnostic delay and inappropriate treatment.

## Figures and Tables

**Table 1:** Overview and clinical relevance of the reviewed studies. EFS: event-free survival; OS: overall survival; SE: standard error.

Study	Study Population	Main Topic	Type of Tumor	Tumor Location	Diagnostic and Treatment Strategies	Outcomes
**Eaton et al.** [20]	Case report	Diagnostic challenges of high-grade UPS	Primary bone tumor	Tibia	MRI with contrast, needle core biopsy, CT for metastasis. Treatment: embolisation of 3 arterial branches, R0 surgical resection, and soft tissue reconstruction.	Patient discharged after 14 days; no long-term follow-up performed.
**Bielack et al.** [21]	132 UPS 25 of tibia analysed retrospectively	Multi-decade analysis of 132 UPS patients	Primary bone tumor	Extremities (24 were tibial cases, 1 of which of the distal tibia)	Chemotherapy (doxorubicin, cisplatin, etc.) and surgery (81% limb-salvage, 15% amputation, 4% rotationplasty).	1, 2, 5, 10 yrs EFS 95% (SE 2%), 86% (3%), 70% (4%), 64% (5%) and OS 88% (3%), 78% (4%), 63% (5%), 55% (5%). Poor prognosis linked to age ≥ 40 and metastasis.
**Gonzalez et al.** [15]	386 cases analysed retrospectively	Large retrospective study on predictors of survival	Soft tissue tumor	Extremities (general)	Retrospective analysis focusing on tumor size and metastasis.	Tumor size ≥ 7 cm and metastasis to lungs/lymph nodes were major predictors of poor outcomes.
**Zhang et al.** [22]	166 cases analysed retrospectively	Retrospective study on survival markers	Soft tissue tumor	Trunk and extremities	55-month mean follow-up analysis.	Identified tumor size ≥ 5 cm and R1/R2 resection margins as markers for poor survival.
**Huff et al.** [23]	Case report	Complex geriatric case with sciatic involvement	Soft tissue tumor	Thigh (encircling sciatic nerve)	X-ray, MRI (revealed 13.7 × 12.3 × 38.6 cm mass), needle biopsy, PET-CT. Treatment: Palliative radiotherapy and chemotherapy.	Poor outcome: patient departed 4 months after diagnosis (6 months after symptoms began).
**Alpert et al.** [24]	Case report	Rapidly growing mass presentation	Soft tissue tumor	Anterior Calf	Initial aspiration and subsequent reconstructive surgery with anterolateral thigh free flap and split-thickness skin graft	Disease-free 15 months after surgery.

## Data Availability

The dataset supporting the conclusions of this review is available upon request to the corresponding author.
